# Supplementary material for: Reliability in long-term clinical studies of disease-modifying therapies for relapsing-remitting multiple sclerosis: A systematic review
Source: PLoS One. 2020 Jun 16;15(6):e0231722. doi: 10.1371/journal.pone.0231722 (PMC7297314; doi:10.1371/journal.pone.0231722)
Supplement: S1 Appendix — (DOC) [file pone.0231722.s001.doc]

**Reliability in long-term clinical studies of disease-modifying therapies for relapsing-remitting multiple sclerosis: A systematic review**

Rosa C Lucchetta ^a^, Letícia P Leonart ^a^, Marcus V M Gonçalves ^b^, Jefferson Becker ^c^, Roberto Pontarolo ^a^, Fernando Fernandez-Llimós ^d^, Astrid Wiens ^a^.

^a^ Graduate Program in Pharmaceutical Sciences, Federal University of Paraná, Brazil.

^b^ Universidade da região de Joinville (UNIVILLE), Brazil.

^c^ Brain Institute and School of Medicine, Pontifical Catholic University of Rio Grande do Sul, Brazil.

^d^ Research Institute for Medicines (iMed.ULisboa), Department of Social Pharmacy, Faculty of Pharmacy, University of Lisbon, Portugal.

**Correspondence**

Astrid Wiens, Graduate Program in Pharmaceutical Sciences, Federal University of Paraná, Brazil, Prof. Lothário Meissner, 632, Curitiba, Paraná, Brazil, [astrid@ufpr.br](mailto:astrid@ufpr.br), +55 41 3360-4161.

**Appendice**

[1- PRISMA checklist 2](#__RefHeading___Toc24295809)

[2- Search strategies 5](#__RefHeading___Toc24295810)

[3- Records excluded during the eligibility phase 6](#__RefHeading___Toc24295811)

[4- Supplemental characteristics of the included long-term studies in systematic review 10](#__RefHeading___Toc24295812)

[5- Risk of bias 11](#__RefHeading___Toc24295813)

[6- Safety outcomes in studies of long-term for relapsing-remitting multiple sclerosis. 12](#__RefHeading___Toc24295814)

[7- References of included long-term studies 16](#__RefHeading___Toc24295815)

# PRISMA checklist

| **Section/topic** | **#** | **Checklist item** | **Reported on page #** |
| --- | --- | --- | --- |
| **TITLE** | | |  |
| Title | 1 | Identify the report as a systematic review, meta-analysis, or both. | 1 |
| **ABSTRACT** | | |  |
| Structured summary | 2 | Provide a structured summary including, as applicable: background; objectives; data sources; study eligibility criteria, participants, and interventions; study appraisal and synthesis methods; results; limitations; conclusions and implications of key findings; systematic review registration number. | 1 |
| **INTRODUCTION** | | |  |
| Rationale | 3 | Describe the rationale for the review in the context of what is already known. | 2 |
| Objectives | 4 | Provide an explicit statement of questions being addressed with reference to participants, interventions, comparisons, outcomes, and study design (PICOS). | 2 |
| **METHODS** | | |  |
| Protocol and registration | 5 | Indicate if a review protocol exists, if and where it can be accessed (e.g., Web address), and, if available, provide registration information including registration number. | 4 |
| Eligibility criteria | 6 | Specify study characteristics (e.g., PICOS, length of follow-up) and report characteristics (e.g., years considered, language, publication status) used as criteria for eligibility, giving rationale. | 4 |
| Information sources | 7 | Describe all information sources (e.g., databases with dates of coverage, contact with study authors to identify additional studies) in the search and date last searched. | 4 |
| Search | 8 | Present full electronic search strategy for at least one database, including any limits used, such that it could be repeated. | S2 |
| Study selection | 9 | State the process for selecting studies (i.e., screening, eligibility, included in systematic review, and, if applicable, included in the meta-analysis). | 5 |
| Data collection process | 10 | Describe method of data extraction from reports (e.g., piloted forms, independently, in duplicate) and any processes for obtaining and confirming data from investigators. | 5 |
| Data items | 11 | List and define all variables for which data were sought (e.g., PICOS, funding sources) and any assumptions and simplifications made. | 5 |
| Risk of bias in individual studies | 12 | Describe methods used for assessing risk of bias of individual studies (including specification of whether this was done at the study or outcome level), and how this information is to be used in any data synthesis. | 5 |
| Summary measures | 13 | State the principal summary measures (e.g., risk ratio, difference in means). | 5 |
| Synthesis of results | 14 | Describe the methods of handling data and combining results of studies, if done, including measures of consistency (e.g., I^2^) for each meta-analysis. | NA |
| Risk of bias across studies | 15 | Specify any assessment of risk of bias that may affect the cumulative evidence (e.g., publication bias, selective reporting within studies). | NA |
| Additional analyses | 16 | Describe methods of additional analyses (e.g., sensitivity or subgroup analyses, meta-regression), if done, indicating which were pre-specified. | NA |
| **RESULTS** | | |  |
| Study selection | 17 | Give numbers of studies screened, assessed for eligibility, and included in the review, with reasons for exclusions at each stage, ideally with a flow diagram. | 6 |
| Study characteristics | 18 | For each study, present characteristics for which data were extracted (e.g., study size, PICOS, follow-up period) and provide the citations. | 7 |
| Risk of bias within studies | 19 | Present data on risk of bias of each study and, if available, any outcome level assessment (see item 12). | S4 |
| Results of individual studies | 20 | For all outcomes considered (benefits or harms), present, for each study: (a) simple summary data for each intervention group (b) effect estimates and confidence intervals, ideally with a forest plot. | 11 |
| Synthesis of results | 21 | Present results of each meta-analysis done, including confidence intervals and measures of consistency. | NA |
| Risk of bias across studies | 22 | Present results of any assessment of risk of bias across studies (see Item 15). | NA |
| Additional analysis | 23 | Give results of additional analyses, if done (e.g., sensitivity or subgroup analyses, meta-regression [see Item 16]). | NA |
| **DISCUSSION** | | |  |
| Summary of evidence | 24 | Summarize the main findings including the strength of evidence for each main outcome; consider their relevance to key groups (e.g., healthcare providers, users, and policy makers). | 15 |
| Limitations | 25 | Discuss limitations at study and outcome level (e.g., risk of bias), and at review-level (e.g., incomplete retrieval of identified research, reporting bias). | 18 |
| Conclusions | 26 | Provide a general interpretation of the results in the context of other evidence, and implications for future research. | 18 |
| **FUNDING** | | |  |
| Funding | 27 | Describe sources of funding for the systematic review and other support (e.g., supply of data); role of funders for the systematic review. | Submission system |

*From:*  Moher D, Liberati A, Tetzlaff J, Altman DG, The PRISMA Group (2009). Preferred Reporting Items for Systematic Reviews and Meta-Analyses: The PRISMA Statement. PLoS Med 6(7): e1000097. doi:10.1371/journal.pmed1000097

For more information, visit: **www.prisma-statement.org**.

# Search strategies

**PubMed**

|  | ***Query*** |
| --- | --- |
| ***#1*** | ("Multiple Sclerosis, Relapsing-Remitting"[MESH] OR “Multiple Sclerosis”[TIAB] OR RRMS[TIAB] OR RMS[TIAB]) |
| ***#2*** | (natalizumab[MESH] OR natalizumab[TIAB] OR alemtuzumab[TIAB] OR “Interferon beta-1a”[MESH] OR “Interferon beta-1a”[TIAB] OR avonex[TIAB] OR rebif[TIAB] OR Interferon beta-1b[MESH] OR “Interferon beta-1b”[TIAB] OR betaferon[TIAB] OR extavia[TIAB] OR “Fingolimod Hydrochloride”[MESH] OR fingolimod[TIAB] OR “Dimethyl Fumarate”[MESH] OR “dimethyl fumarate”[TIAB] OR “Glatiramer Acetate”[MESH] OR glatiramer[TIAB] OR ocrelizumab[TIAB] OR azathioprine[MESH] OR azathioprine[TIAB] OR peginterferon[TIAB] OR teriflunomide[Supplementary Concept] OR teriflunomide[TIAB] OR cladribine[MESH] OR cladribine[TIAB]) |
| ***#3*** | ((long-term[TIAB] OR extension[TIAB] OR year*[TI] OR follow-up[TI])) |
| ***#4*** | ((animals[MH:noexp] NOT (animals[MH:noexp] AND humans[MH]))) |
| ***#5*** | ((letter[PT] OR editorial[PT] OR historical article[PT] OR review[PT])) |
| ***#6*** | **#1 and #2 and #3 not #4 not #5** |

**Scopus**

|  | ***Query*** |
| --- | --- |
| ***#1*** | TITLE-ABS-KEY((relapsing-remitting AND "Multiple Sclerosis") OR “Multiple Sclerosis” OR RRMS OR RMS) |
| ***#2*** | TITLE-ABS-KEY (natalizumab OR alemtuzumab OR "Interferon beta-1a" OR avonex OR rebif OR "Interferon beta-1b" OR betaferon OR extavia OR fingolimod OR "Dimethyl Fumarate" OR "Glatiramer Acetate" OR glatiramer OR ocrelizumab OR azathioprine OR teriflunomide OR cladribine) |
| ***#3*** | TITLE-ABS-KEY(long-term OR extension OR year* OR follow-up) |
| ***#4*** | TITLE-ABS-KEY((animals AND NOT (animals AND humans)) |
| ***#5*** | DOCTYPE(le OR ed OR re) |
| ***#6*** | INDEX(Medline) |
| ***#7*** | **#1 and #2 and #3 AND NOT #4 AND NOT #5 AND NOT #6** |

# Records excluded during the eligibility phase

| **Population (n = 6)** |
| --- |
| Gold, R.; Giovannoni, G.; Phillips, J. T.; Fox, R. J.; Zhang, A.; Marantz, J. L. Sustained Effect of Delayed-Release Dimethyl Fumarate in Newly Diagnosed Patients with Relapsing-Remitting Multiple Sclerosis: 6-Year Interim Results From an Extension of the DEFINE and CONFIRM Studies 2016 Neurol Ther 5 1 45-57 10.1007/s40120-016-0042-8  Comi, G.; Freedman, M. S.; Kappos, L.; Olsson, T. P.; Miller, A. E.; Wolinsky, J. S.; O'Connor, P. W.; Benamor, M.; Dukovic, D.; Truffinet, P.; Leist, T. P. Pooled safety and tolerability data from four placebo-controlled teriflunomide studies and extensions 2016 Mult Scler Relat Disord 5 97-104 10.1016/j.msard.2015.11.006  Clanet, M.; Kappos, L.; Hartung, H. P.; Hohlfeld, R. Interferon beta-1a in relapsing multiple sclerosis: four-year extension of the European IFNbeta-1a Dose-Comparison Study 2004 Mult Scler 10 2 139-44 10.1191/1352458504ms990oa  O'Connor, P.; Comi, G.; Freedman, M. S.; Miller, A. E.; Kappos, L.; Bouchard, J. P.; Lebrun-Frenay, C.; Mares, J.; Benamor, M.; Thangavelu, K.; Liang, J.; Truffinet, P.; Lawson, V. J.; Wolinsky, J. S. Long-term safety and efficacy of teriflunomide: Nine-year follow-up of the randomized TEMSO study 2016 Neurology 86 10 920-30 10.1212/wnl.0000000000002441  Confavreux, C.; Li, D. K.; Freedman, M. S.; Truffinet, P.; Benzerdjeb, H.; Wang, D.; Bar-Or, A.; Traboulsee, A. L.; Reiman, L. E.; O'Connor, P. W. Long-term follow-up of a phase 2 study of oral teriflunomide in relapsing multiple sclerosis: safety and efficacy results up to 8.5 years 2012 Mult Scler 18 9 1278-89 10.1177/1352458512436594  Comi, G.; O'Connor, P.; Montalban, X.; Antel, J.; Radue, E. W.; Karlsson, G.; Pohlmann, H.; Aradhye, S.; Kappos, L. Phase II study of oral fingolimod (FTY720) in multiple sclerosis: 3-year results 2010 Mult Scler 16 2 197-207 10.1177/1352458509357065 |
| **Intervention and/ or control (n = 6)** |
| Paolicelli, D.; Lucisano, G.; Manni, A.; Avolio, C.; Bonavita, S.; Brescia Morra, V.; Capobianco, M.; Cocco, E.; Conte, A.; De Luca, G.; De Robertis, F.; Gasperini, C.; Gatto, M.; Gazzola, P.; Lus, G.; Iaffaldano, A.; Iaffaldano, P.; Maimone, D.; Mallucci, G.; Maniscalco, G. T.; Marfia, G. A.; Patti, F.; Pesci, I.; Pozzilli, C.; Rovaris, M.; Salemi, G.; Salvetti, M.; Spitaleri, D.; Totaro, R.; Zaffaroni, M.; Comi, G.; Amato, M. P.; Trojano, M. Retrospectively acquired cohort study to evaluate the long-term impact of two different treatment strategies on disability outcomes in patients with relapsing multiple sclerosis (RE.LO.DI.MS): data from the Italian MS Register 2019 J Neurol 10.1007/s00415-019-09531-6  Ebers GC, Traboulsee A, Li D, et al. Analysis of clinical outcomes according to original treatment groups 16 years after the pivotal IFNB-1b trial 2010 J Neurol Neurosurg Psychiatry 81 907–912  Saida, T.; Kira, J.; Ueno, Y.; Harada, N.; Hirakata, T. Long-term efficacy and safety of intramuscular interferon beta-1a: Randomized postmarketing trial of two dosing regimens in Japanese patients with relapsing-remitting multiple sclerosis 2016 Mult Scler Relat Disord 7 102-8 10.1016/j.msard.2016.02.002  Uitdehaag, B.; Constantinescu, C.; Cornelisse, P.; Jeffery, D.; Kappos, L.; Li, D.; Sandberg-Wollheim, M.; Traboulsee, A.; Verdun, E.; Rivera, V. Impact of exposure to interferon beta-1a on outcomes in patients with relapsing-remitting multiple sclerosis: exploratory analyses from the PRISMS long-term follow-up study 2011 Ther Adv Neurol Disord 4 1 3-14 10.1177/1756285610391693  O'Connor, P.; Goodman, A.; Kappos, L.; Lublin, F.; Polman, C.; Rudick, R. A.; Hauswirth, K.; Cristiano, L. M.; Forrestal, F.; Duda, P. Long-term safety and effectiveness of natalizumab redosing and treatment in the STRATA MS Study 2014 Neurology 83 1 78-86 10.1212/wnl.0000000000000541  Ghezzi, A.; Chitnis, T.; K. Laflamme A; Meinert, R.; Haring, D. A.; Pohl, D. Long-Term Effect of Immediate Versus Delayed Fingolimod Treatment in Young Adult Patients with Relapsing-Remitting Multiple Sclerosis: Pooled Analysis from the FREEDOMS/FREEDOMS II Trials 2019 Neurol Ther 10.1007/s40120-019-0146-z |
| **Outcomes (n = 13)** |
| Brochet, B. [Long-term effects of glatiramer acetate in multiple sclerosis] 2008 Rev Neurol (Paris) 164 11 917-26 10.1016/j.neurol.2008.02.045  Kappos, L.; Kuhle, J.; Multanen, J.; Kremenchutzky, M.; Verdun di Cantogno, E.; Cornelisse, P.; Lehr, L.; Casset-Semanaz, F.; Issard, D.; Uitdehaag, B. M. Factors influencing long-term outcomes in relapsing-remitting multiple sclerosis: PRISMS-15 2015 J Neurol Neurosurg Psychiatry 86 11 1202-7 10.1136/jnnp-2014-310024  Rinaldi, F.; Perini, P.; Atzori, M.; Favaretto, A.; Seppi, D.; Gallo, P. Disease-modifying drugs reduce cortical lesion accumulation and atrophy progression in relapsing-remitting multiple sclerosis: results from a 48-month extension study 2015 Mult Scler Int 2015 369348 10.1155/2015/369348  Goodin, D. S.; Reder, A. T.; Ebers, G. C.; Cutter, G.; Kremenchutzky, M.; Oger, J.; Langdon, D.; Rametta, M.; Beckmann, K.; DeSimone, T. M.; Knappertz, V. Survival in MS: a randomized cohort study 21 years after the start of the pivotal IFNbeta-1b trial 2012 Neurology 78 17 1315-22 10.1212/WNL.0b013e3182535cf6  Singer, B.; Wray, S.; Miller, T.; Cascione, M.; Gupta, A.; Pardo, G.; Watsky, E.; Hayward, B.; Mercer, B.; Dangond, F. Patient-rated ease of use and functional reliability of an electronic autoinjector for self-injection of subcutaneous interferon beta-1a for relapsing multiple sclerosis 2012 Mult Scler Relat Disord 1 2 87-94 10.1016/j.msard.2011.11.002  Durelli, L.; Barbero, P.; Bergui, M.; Versino, E.; Bassano, M. A.; Verdun, E.; Rivoiro, C.; Ferrero, C.; Picco, E.; Ripellino, P.; Giuliani, G.; Montanari, E.; Clerico, M. MRI activity and neutralising antibody as predictors of response to interferon beta treatment in multiple sclerosis 2008 J Neurol Neurosurg Psychiatry 79 6 646-51 10.1136/jnnp.2007.130229  Lugaresi, A.; Durastanti, V.; Gasperini, C.; Lai, M.; Pozzilli, C.; Orefice, G.; Sotgiu, S.; Pucci, E.; Ardito, B.; Millefiorini, E. Safety and tolerability in relapsing-remitting multiple sclerosis patients treated with high-dose subcutaneous interferon-beta by Rebiject autoinjection over a 1-year period: the CoSa study 2008 Clin Neuropharmacol 31 3 167-72 10.1097/wnf.0b013e3181571a8e  Brown, M. G.; Kirby, S.; Skedgel, C.; Fisk, J. D.; Murray, T. J.; Bhan, V.; Sketris, I. S. How effective are disease-modifying drugs in delaying progression in relapsing-onset MS? 2007 Neurology 69 15 1498-507 10.1212/01.wnl.0000271884.11129.f3  Fisher, E.; Rudick, R. A.; Cutter, G.; Baier, M.; Miller, D.; Weinstock-Guttman, B.; Mass, M. K.; Dougherty, D. S.; Simonian, N. A. Relationship between brain atrophy and disability: an 8-year follow-up study of multiple sclerosis patients 2000 Mult Scler 6 6 373-7 10.1177/135245850000600602  Rovaris, M.; Comi, G.; Rocca, M. A.; Valsasina, P.; Ladkani, D.; Pieri, E.; Weiss, S.; Shifroni, G.; Wolinsky, J. S.; Filippi, M. Long-term follow-up of patients treated with glatiramer acetate: a multicentre, multinational extension of the European/Canadian double-blind, placebo-controlled, MRI-monitored trial 2007 Mult Scler 13 4 502-8 10.1177/1352458506070704  Healy, B. C.; Glanz, B. I.; Zurawski, J. D.; Mazzola, M.; Chitnis, T.; Weiner, H. L. Long-term follow-up for multiple sclerosis patients initially treated with interferon-beta and glatiramer acetate 2018 J Neurol Sci 394 127-31 10.1016/j.jns.2018.09.020  Haas, J.; Jeffery, D.; Silva, D.; Meier, D. P.; Meinert, R.; Cohen, J.; Hartung, H. P. Early initiation of fingolimod reduces the rate of severe relapses over the long term: Post hoc analysis from the FREEDOMS, FREEDOMS II, and TRANSFORMS studies 2019 Multiple Sclerosis and Related Disorders 36 10.1016/j.msard.2019.07.011  Cofield, S. S.; Fox, R. J.; Tyry, T.; Salter, A. R.; Campagnolo, D. Disability progression after switching from natalizumab to fingolimod or interferon beta/glatiramer acetate therapies: A NARCOMS analysis 2016 International Journal of MS Care 18 5 230-8 10.7224/1537-2073.2014-113 |
| **Type of study (n = 13)** |
| Kappos, L.; Traboulsee, A.; Constantinescu, C.; Eralinna, J. P.; Forrestal, F.; Jongen, P.; Pollard, J.; Sandberg-Wollheim, M.; Sindic, C.; Stubinski, B.; Uitdehaag, B.; Li, D. Long-term subcutaneous interferon beta-1a therapy in patients with relapsing-remitting MS 2006 Neurology 67 6 944-53 10.1212/01.wnl.0000237994.95410.ce  Ziemssen, T.; Gilgun-Sherki, Y. Sub-analysis of geographical variations in the 2-year observational COPTIMIZE trial of patients with relapsing-remitting multiple sclerosis converting to glatiramer acetate 2015 BMC Neurol 15 189 10.1186/s12883-015-0448-4  Agius M1, Meng X, Chin P, Grinspan A, Hashmonay R Fingolimod therapy in early multiple sclerosis: an efficacy analysis of the TRANSFORMS and FREEDOMS studies by time since first symptom 2014 CNS Neurosci Ther 5 446-51 10.1111/cns.12235  Kira, J.; Itoyama, Y.; Kikuchi, S.; Hao, Q.; Kurosawa, T.; Nagato, K.; Tsumiyama, I.; von Rosenstiel, P.; Zhang-Auberson, L.; Saida, T. Fingolimod (FTY720) therapy in Japanese patients with relapsing multiple sclerosis over 12 months: results of a phase 2 observational extension 2014 BMC Neurol 14 21 10.1186/1471-2377-14-21  Patti, F.; Morra, V. B.; Amato, M. P.; Trojano, M.; Bastianello, S.; Tola, M. R.; Cottone, S.; Plant, A.; Picconi, O. Subcutaneous interferon beta-1a may protect against cognitive impairment in patients with relapsing-remitting multiple sclerosis: 5-year follow-up of the COGIMUS study 2013 PLoS One 8 8 e74111 10.1371/journal.pone.0074111  Lampl, C.; You, X.; Limmroth, V. Weekly IM interferon beta-1a in multiple sclerosis patients over 50 years of age 2012 Eur J Neurol 19 1 142-8 10.1111/j.1468-1331.2011.03460.x  Khatri, B.; Barkhof, F.; Comi, G.; Hartung, H. P.; Kappos, L.; Montalban, X.; Pelletier, J.; Stites, T.; Wu, S.; Holdbrook, F.; Zhang-Auberson, L.; Francis, G.; Cohen, J. A. Comparison of fingolimod with interferon beta-1a in relapsing-remitting multiple sclerosis: a randomised extension of the TRANSFORMS study 2011 Lancet Neurol 10 6 520-9 10.1016/s1474-4422(11)70099-0  Ford, C. C.; Johnson, K. P.; Lisak, R. P.; Panitch, H. S.; Shifronis, G.; Wolinsky, J. S. A prospective open-label study of glatiramer acetate: over a decade of continuous use in multiple sclerosis patients 2006 Mult Scler 12 3 309-20 10.1191/135248506ms1318oa  Filippini, G.; Incorvaia, B. Is interferon effective and safe for relapsing-remitting multiple sclerosis? 2002 Neuroepidemiology 21 6 310 10.1159/000065530  Trojano, M.; Zimarore, C.; Caputo, G.; Giuliani, F.; Paolicelli, D.; Avolio, C.; De Roberris, F.; Liguori, M.; Bellacosa, A.; Livrea, P. Post-marketing surveillance of interferon beta treatment in relapsing-remitting multiple sclerosis in southern italy 2001 Neurological Sciences 22 2 214  Knobler, R. L.; Greenstein, J. I.; Johnson, K. P.; Lublin, F. D.; Panitch, H. S.; Conway, K.; Grant-Gorsen, S. V.; Muldoon, J.; Marcus, S. G.; Wallenberg, J. C.; et al., Systemic recombinant human interferon-beta treatment of relapsing-remitting multiple sclerosis: pilot study analysis and six-year follow-up 1993 J Interferon Res 13 5 333-40 10.1089/jir.1993.13.333  Boster, A.; Nicholas, J.; Wu, N.; Yeh, W. S.; Fay, M.; Edwards, M.; Huang, M. Y.; Lee, A. Comparative Effectiveness Research of Disease-Modifying Therapies for the Management of Multiple Sclerosis: Analysis of a Large Health Insurance Claims Database 2017 Neurology and Therapy 6 1 91-102 10.1007/s40120-017-0064-x  Giovannoni, G.; Soelberg Sorensen, P.; Cook, S.; Rammohan, K. W.; Rieckmann, P.; Comi, G.; Dangond, F.; Hicking, C.; Vermersch, P. Efficacy of Cladribine Tablets in high disease activity subgroups of patients with relapsing multiple sclerosis: A post hoc analysis of the CLARITY study 2019 Multiple Sclerosis Journal 25 6 819-27 10.1177/1352458518771875 |

# Supplemental characteristics of the included long-term studies in systematic review

| **Study** | **Diagnostic criteria** | **Sponsor** |
| --- | --- | --- |
| **ADVANCE/ ATTAIN** ^1^ | McDonald 2005 | Biogen (Cambridge, MA, USA) |
| **CAMMS223** ^2,3^ | McDonald 2001 | Genzyme and Bayer Schering Pharma |
| **CARE-MS I** ^4^ | McDonald 2005 | Sanofi and Bayer HealthCare Pharmaceuticals. |
| **CARE-MS II** ^5^ | McDonald 2005 | Genzyme (SANOFI) and Bayer Schering Pharma. |
| **CLARITY** ^6^ | McDonald 2001 | Merck Serono S.A. |
| **CMSSG** ^7–9^ | Poser 1983 | FDA and National Multiple Sclerosis Society |
| **CombiRx** ^10,11^ | McDonald 2001 | H National Institute of Neurological Disorders and Stroke |
| **ENDORSE** ^12^ | McDonald 2005 | Biogen |
| **FREEDOMS** ^13^ | McDonald 2005 | Novartis |
| **GALA** ^14^ | McDonald 2005 | Teva Ltd. |
| **Moccia, 2018** ^15^ | McDonald 2001 | No |
| **Onesti, 2003** ^16^ | Poser 1983 | NR |
| **OWIMS** ^17^ | Poser 1983 | Serono International AS |
| **Patti, 2006** ^18^ | Poser 1983 | No |
| **PRISMS** ^19–21^ | Poser 1983 | Ares-Serono International AS |
| **Río, 2005** ^22^ | Poser 1983 | Red CIEN |
| **Ruggieri, 2003** ^23^ | NR | NR |
| **Saida** ^24^ | McDonald 2005 | Novartis Pharma KK and Mitsubishi Tanabe Pharma Corporation |
| **TRANSFORMS** ^25^ | McDonald 2005 | Novartis Pharma |

NR: not reported.

# Risk of bias

| **Study** | **Confounding** | **Selection of participants into the study** | **Classification of interventions** | **Deviations from intended interventions** | **Missing data** | **Measurement of outcomes** | **Selections of the reported result** | **Overall bias** |
| --- | --- | --- | --- | --- | --- | --- | --- | --- |
| ATTAIN | Serious | Serious | Low | Low | Serious | Serious | Low | Serious |
| CAMMS223 | Serious | Serious | Low | Low | Serious | Serious | Moderate | Serious |
| CARE-MS I | NI | NI | NI | NI | NI | NI | NI | Critical |
| CARE-MS II | NI | NI | NI | NI | NI | NI | NI | Critical |
| CLARITY | Serious | Serious | Low | Low | Serious | Low | Moderate | Serious |
| CMSSG | Serious | Serious | Low | Low | Moderate | Low | Low | Serious |
| CombiRx | Low | Low | Low | Low | Serious | Low | Low | Serious |
| ENDORSE | Serious | Serious | Low | Low | Serious | Low | Low | Serious |
| FREEDOMS | Serious | Serious | Low | Low | Serious | Low | Low | Serious |
| GALA | NI | NI | NI | NI | NI | NI | NI | Critical |
| Moccia, 2018 | Moderate | Serious | Low | Low | Serious | Serious | Moderate | Serious |
| Onesti, 2003 | Serious | Serious | Low | Low | Serious | Moderate | Serious | Serious |
| OWIMS | Serious | Serious | Low | Low | Moderate | Low | Low | Serious |
| Patti, 2006 | Serious | Moderate | Low | Low | Serious | Serious | Moderate | Serious |
| PRISMS | Serious | Serious | Low | Low | Serious | Low | Low | Serious |
| Río, 2005 | Serious | Moderate | Low | Low | Serious | Serious | Moderate | Serious |
| Ruggieri, 2003 | Serious | Moderate | Low | Low | Moderate | Serious | Moderate | Serious |
| Saida | NI | NI | NI | NI | NI | NI | NI | Critical |
| TRANSFORMS | Serious | Serious | Low | Low | Serious | Low | Low | Serious |

# Safety outcomes in studies of long-term for relapsing-remitting multiple sclerosis.

|  |  | **Mid-Term** | | **Long-Term** | | | |
| --- | --- | --- | --- | --- | --- | --- | --- |
| **Alternative** | **Study** | **Follow-up, months** | **Patient (%) [Sample]** | | **Follow-up, months** | | **Patient (%) [Sample]** |
| **Discontinuation due to adverse event** | | | | | | | |
| **ALE12** | CARE-MS I | 0 – 24 | 5 (1.33) [376] | 24 – 36  36 – 48  48 – 60 | | 0 (0.00) [360]  0 (0.00) [344]  0 (0.00) [340] | |
|  | CARE-MS II | 0 – 24 | 14 (3.22) [435] | 24 – 36  36 – 48  48 – 60 | | 1 (2.43) [412]  3 (0.78) [387]  2 (0.75) [267] | |
|  | CAMMS23 | - | - | 0 – 32 | | 2 (1.86) [108] | |
|  | CAMMS23 | - | - | 0 – 60 | | 5 (4.63) [108] | |
| **ALE24** | CARE-MS II | 0 – 24 | 6 (3.73) [161] | - | | - | |
|  | CAMMS23 | - | - | 0 – 32 | | 1 (0.93) [108] | |
|  | CAMMS23 | - | - | 0 – 60 | | 2 (1.86) [108] | |
| **CLA3.5** | CLARITY | 0 – 24 | 15 (3.46) [433] | 24 – 48 | | 26 (14.00) [186] | |
| **BG240BID** | CONFIRM | 0 – 24 | 44 (12.26) [359] | - | | - | |
|  | DEFINE | 0 – 24 | 65 (15.85) [410] | - | | - | |
|  | ENDORSE | - | - | 24 – 36 | | 28 (5.59) [501] | |
|  | ENDORSE | - | - | 36 – 48 | | 16 (13.56) [118] § | |
|  | ENDORSE | - | - | 48 – 60 | | 42 (16.87) [249] ‡ | |
| **BG240TID** | CONFIRM | 0 – 24 | 41 (11.92) [344] | - | | - | |
|  | DEFINE | 0 – 24 | 68 (16.35) [416] | - | | - | |
|  | ENDORSE | - | - | 24 – 36 | | 36 (7.19) [501] | |
|  | ENDORSE | - | - | 36 – 48 | | 31 (26.06) [119] § | |
|  | ENDORSE | - | - | 48 – 60 | | 41 (16.54) [248] ‡ | |
| **FING0.5QD** | Saida 2017 | - | - | 0 – 36 | | 7 (14.90) [47] | |
|  | GIMN | 0 – 6 | 8 (3.48) [230] | - | | - | |
|  | TRANSFORMS | 0 – 12 | 24 (5.59) [429] | - | | - | |
|  | FREEDOMS | 0 – 24 | 32 (7.53) [425] | 24 – 48 | | 15 (4.54) [331] | |
|  | FREEDOMS II | 0 – 24 | 66 (18.44) [358] | - | | - | |
| **FING1.25QD** | Saida 2017 | - | - | 0 – 36 | | 2 (4.30) [46] | |
|  | FREEDOMS | 0 – 24 | 61 (14.22) [429] | 24 – 48 | | 16 (5.54) [289] | |
|  | FREEDOMS II | 0 – 24 | 72 (19.46) [370] | - | | - | |
|  | TRANSFORMS | 0 – 12 | 42 (10.00) [420] | 12 – 54 | | 36 (8.40) [429] | |
| **GA20QD** | GLACIER | 0 – 4 | 0 (0.00) [101] | - | | - | |
|  | GIMN | 0 – 6 | 3 (7.50) [40] | - | | - | |
|  | GATE | 0 – 9 | 4 (1.12) [357] | - | | - | |
|  | ECGA | 0 – 9 | 3 (2.52) [119] | - | | - | |
|  | CONFIRM | 0 – 24 | 35 (9.97) [351] | - | | - | |
|  | CMSSG | 0 – 24 | 4 (3.20) [125] | - | | - | |
|  | BEYOND | 0 – 24 | 8 (1.79) [448] | - | | - | |
|  | Boiko, 2018 | 0 – 12 | 1 (1.59) [63] | - | | - | |
|  | Cohen, 2007 | 0 – 9 | 1 (2.27) [44] | - | | - | |
|  | CORAL | 0 – 12 | 28 (4.78) [586] | - | | - | |
|  | COMBIRX | - | - | 0 – 32 | | 11 (4.25) [259] | |
| **IFNA22TIW** | OWIMS | 0 – 12 | 1 (1.05) [95] | 0 – 36 | | 3 (3.16) [95] | |
|  | PRISMS | 0 – 24 | 6 (3.17) [189] | 0 – 48 | | 9 (4.77) [189] | |
|  | Ruggieri, 2003 | - | - | 0 – 60 | | 5 (27.78) [18] | |
| **IFNA30QW** | TRANSFORMS | 0 – 12 | 16 (3.71) [431] | - | | - | |
|  | MSCRG | 0 – 24 | 7 (4.43) [158] | - | | - | |
|  | INCOMIN | 0 – 24 | 1 (1.09) [92] | - | | - | |
|  | EVIDENCE | 0 – 24 | 18 (5.33) [338] | - | | - | |
|  | BRAVO | 0 – 24 | 26 (5.88) [442] | - | | - | |
|  | Patti, 2006 | - | - | 0 – 72 | | 0 (0.00) [62] | |
|  | CombiRx | - | - | 0 – 32 | | 17 (6.80) [250] | |
|  | Ruggieri, 2003 | - | - | 0 – 60 | | 4 (10.53) [38] | |
|  | Ruggieri, 2003 | - | - | 0 – 60 | | 3 (30.00) [10] ∥ | |
| **IFNA44TIW** | OWIMS | 0 – 12 | 5 (5.10) [98] | 0 – 36 | | 10 (10.21) [98] | |
|  | Kappos 2011 | 0 – 12 | 1 (1.85) [54] | - | | - | |
|  | GALA | 0 – 12 | 29 (3.08) [943] | - | | - | |
|  | GIMN | 0 – 6 | 2 (7.14) [28] | - | | - | |
|  | EVIDENCE | 0 – 24 | 19 (5.60) [339] | - | | - | |
|  | PRISMS | 0 – 24 | 9 (4.89) [184] | 0 – 48 | | 18 (9.79) [184] | |
|  | CARE-MS I | 0 – 24 | 11 (5.88) [187] | - | | - | |
|  | CARE-MS II | 0 – 24 | 15 (7.43) [202] | - | | - | |
|  | OPERA I | 0 – 24 | 26 (6.36) [409] | - | | - | |
|  | OPERA II | 0 – 24 | 25 (6.00) [417] | - | | - | |
|  | REGARD | 0 – 24 | 23 (5.96) [386] | - | | - | |
|  | CAMMS23 | - | - | 0 – 32 | | 13 (12.15) [107] | |
|  | CAMMS23 | - | - | 0 – 60 | | 13 (12.15) [107] | |
| **IFNB250EOD** | INCOMIN | 0 – 24 | 5 (5.21) [96] | - | | - | |
|  | BEYOND | 0 – 24 | 13 (1.45) [897] | - | | - | |
|  | Patti, 2006 | - | - | 0 – 72 | | 4 (6.25) [64] | |
|  | Ruggieri, 2003 | - | - | 0 – 60 | | 16 (28.58) [56] | |
| **PIFN125Q2W** | ADVANCE | 0 – 24 | 41 (6.00) [740] | 24 – 72 | | 26 (5.00) [547] | |
| **PIFN125Q4W** | ADVANCE | 0 – 24 | 42 (6.00) [728] | 24 – 72 | | 18 (3.00) [529] | |
| **Serious adverse event** | | | | | | | |
| **ALE12** | CARE-MS I | 0 – 24 | 69 (18.35) [376] | 24 – 36  36 – 48  48 – 60 | | 36 (10.00) [360]  29 (8.43) [344]  17 (5.00) [340] | |
|  | CARE-MS II | 0 – 24 | 85 (19.54) [435] | 24 – 36  36 – 48  48 – 60 | | 39 (9.50) [412]  53 (13.70) [387]  36 (9.80) [367] | |
|  | CAMMS23 | - | - | 0 – 32 | | 24 (22.23) [108] | |
|  | CAMMS23 | - | - | 0 – 60 | | 30 (27.78) [108] | |
| **ALE24** | CARE-MS II | 0 – 24 | 30 (18.63) [161] | - | | - | |
|  | CAMMS23 | - | - | 0 – 32 | | 27 (25.00) [108] | |
|  | CAMMS23 | - | - | 0 – 60 | | 33 (30.56) [108] | |
| **BG240BID** | CONFIRM | 0 – 24 | 61 (16.99) [359] | - | | - | |
|  | DEFINE | 0 – 24 | 74 (18.05) [410] | - | | - | |
|  | ENDORSE | - | - | 24 – 36 | | 109 (21.76) [501] | |
|  | ENDORSE | - | - | 36 – 48 | | 19 (16.11) [118] § | |
|  | ENDORSE | - | - | 48 – 60 | | 59 (23.70) [249] ‡ | |
| **BG240TID** | CONFIRM | 0 – 24 | 54 (15.70) [344] | - | | - | |
|  | DEFINE | 0 – 24 | 65 (15.63) [416] | - | | - | |
|  | ENDORSE | - | - | 24 – 36 | | 124 (24.76) [501] | |
|  | ENDORSE | - | - | 36 – 48 | | 23 (19.33) [119] § | |
|  | ENDORSE | - | - | 48 – 60 | | 40 (16.13) [248] ‡ | |
| **CLA3.5** | CLARITY | 0 – 24 | 36 (8.31) [433] | 24 – 48 | | 25 (13.40) [186] | |
| **FING0.5QD** | Saida 2017 | 0 – 6 | - | 0 – 36 | | 8 (17.00) [47] | |
|  | TRANSFORMS | 0 – 12 | 30 (6.99) [429] | 0 – 54 | | 55 (15.40) [356] † | |
|  | FREEDOMS | 0 – 24 | 43 (10.12) [425] | 24 – 48 | | 31 (9.37) [331] | |
|  | FREEDOMS II | 0 – 24 | 53 (14.80) [358] | - | | - | |
| **FING1.25QD** | Saida 2017 | 0 – 6 | - | 0 – 36 | | 5 (10.90) [46] | |
|  | FREEDOMS | 0 – 24 | 51 (11.89) [429] | 24 – 48 | | 31 (10.73) [289] | |
|  | FREEDOMS II | 0 – 24 | 53 (14.32) [370] | - | | - | |
|  | TRANSFORMS | 0 – 12 | 45 (10.71) [420] | - | | - | |
| **GA20QD** | CONFIRM | 0 – 24 | 60 (11.89) [351] | - | | - | |
|  | Boiko 2018 | 0 – 12 | 3 (4.76) [63] | - | | - | |
|  | GATE | 0 – 9 | 17 (4.76) [357] | - | | - | |
|  | ECGA | 0 – 9 | 10 (8.40) [119] | - | | - | |
|  | Cohen 2007 | 0 – 9 | 1 (2.27) [44] | - | | - | |
|  | CORAL | 0 – 12 | 25 (4.27) [586] | - | | - | |
|  | CMMSG | - | - | 0 – 96 | | 0 (0.00) [142] | |
|  | COMBIRX | - | - | 0 – 32 | | 30 (11.59) [259] | |
| **IFNA22TIW** | OWIMS | 0 – 12 | 5 (5.26) [95] | - | | - | |
|  | PRISMS | - | - | 0 – 48 | | 51 (26.99) [189] | |
| **IFNA30QW** | TRANSFORMS | 0 – 12 | 25 (5.80) [431] | - | | - | |
|  | EVIDENCE | 0 – 24 | 18 (5.34) [337] | - | | - | |
|  | BRAVO | 0 – 24 | 34 (7.69) [442] | - | | - | |
|  | COMBIRX | - | - | 0 – 32 | | 38 (15.20) [250] | |
| **IFNA44TIW** | OWIMS | 0 – 12 | 6 (6.12) [98] | - | | - | |
|  | EVIDENCE | 0 – 24 | 21 (6.19) [339] | - | | - | |
|  | Kappos 2011 | 0 – 12 | 3 (5.56) [54] | - | | - | |
|  | CARE-MS I | 0 – 24 | 27 (14.44) [187] | - | | - | |
|  | CARE-MS II | 0 – 24 | 44 (21.78) [202] | - | | - | |
|  | OPERA I | 0 – 24 | 32 (7.82) [409] | - | | - | |
|  | OPERA II | 0 – 24 | 40 (9.59) [417] | - | | - | |
|  | REGARD | 0 – 24 | 29 (7.61) [381] | - | | - | |
|  | PRISMS | - | - | 0 – 48 | | 30 (16.31) [184] | |
|  | CAMMS23 | - | - | 0 – 32 | | 24 (22.43) [107] | |
|  | CAMMS23 | - | - | 0 – 60 | | 29 (27.11) [107] | |
| **PIFN125Q2W** | ADVANCE | 0 – 24 | 120 (16.00) [740] | 24 – 72 | | 90 (16.00) [547] | |
| **PIFN125Q4W** | ADVANCE | 0 – 24 | 158 (22.00) [728] | 24 – 72 | | 113 (21.00) [529] | |

Results colored in green represent annual incidence and results colored in yellow represent patient presenting event in the complete follow-up; † In at least 2 patients in each group; ‡: switch therapy (placebo 🡪 BG240) with **≥** 3-year in BG240; §: switch therapy (GA 🡪 BG240) with **≥** 3-year in BG240; ∥: switch therapy (IFNB250EOD 🡪 IFNA30QW) with **≥** 3-year in IFNA30QW; ALE12/ ALE24: alemtuzumab, 12 or 24 mg/ day per 5 days and 12 months later per 3 days; BG240BID/ TID: dimethyl fumarate, 240 mg, twice-times daily or three-times daily; FING0.5QD/ 1.25QD: fingolimod, 0.5 or 1.25 mg daily; GA20QD: glatiramer acetate, 20 mg daily; IFNA22TIW/ IFNA44TIW: interferon 1a beta 22 or 44 µg three-times weekly; IFNB250EOD: interferon 1b beta, 250 µg, every other day; IFNA30QW: interferon 1a beta, 30 µg weekly; PLA: placebo.

# References of included long-term studies

| 1. Newsome SD, Scott TF, Arnold DL, Nelles G, Hung S, Cui Y, et al. Long-term outcomes of peginterferon beta-1a in multiple sclerosis: results from the ADVANCE extension study, ATTAIN. Ther Adv Neurol Disord [Internet]. 2018 Jan 28;11:175628641879114. Available from: http://journals.sagepub.com/doi/10.1177/1756286418791143  2. Coles AJ, Fox E, Vladic A, Gazda SK, Brinar V, Selmaj KW, et al. Alemtuzumab more effective than interferon beta-1a at 5-year follow-up of CAMMS223 clinical trial. Neurology. 2012 Apr;78(14):1069–78.  3. Coles AJ, Compston DAS, Selmaj KW, Lake SL, Moran S, Margolin DH, et al. Alemtuzumab vs. interferon beta-1a in early multiple sclerosis. N Engl J Med. 2008 Oct;359(17):1786–801.  4. Havrdova E, Arnold DL, Cohen JA, Hartung H-P, Fox EJ, Giovannoni G, et al. Alemtuzumab CARE-MS I 5-year follow-up: Durable efficacy in the absence of continuous MS therapy. Neurology. 2017 Sep;89(11):1107–16.  5. Coles AJ, Cohen JA, Fox EJ, Giovannoni G, Hartung H-P, Havrdova E, et al. Alemtuzumab CARE-MS II 5-year follow-up: Efficacy and safety findings. Neurology. 2017 Sep;89(11):1117–26.  6. Giovannoni G, Soelberg Sorensen P, Cook S, Rammohan K, Rieckmann P, Comi G, et al. Safety and efficacy of cladribine tablets in patients with relapsing-remitting multiple sclerosis: Results from the randomized extension trial of the CLARITY study. Mult Scler. 2017 Aug;1352458517727603.  7. Johnson KP, Brooks BR, Ford CC, Goodman A, Guarnaccia J, Lisak RP, et al. Sustained clinical benefits of glatiramer acetate in relapsing multiple sclerosis patients observed for 6 years. Copolymer 1 Multiple Sclerosis Study Group. Mult Scler. 2000 Aug;6(4):255–66.  8. Johnson KP, Brooks BR, Cohen JA, Ford CC, Goldstein J, Lisak RP, et al. Extended use of glatiramer acetate (Copaxone) is well tolerated and maintains its clinical effect on multiple sclerosis relapse rate and degree of disability. Copolymer 1 Multiple Sclerosis Study Group. Neurology [Internet]. 1998 Mar;50(3):701–8. Available from: http://www.ncbi.nlm.nih.gov/pubmed/9521260  9. Johnson KP, Ford CC, Lisak RP, Wolinsky JS. Neurologic consequence of delaying glatiramer acetate therapy for multiple sclerosis: 8-year data. Acta Neurol Scand [Internet]. 2004/12/15. 2005 Jan;111(1):42–7. Available from: http://doi.wiley.com/10.1111/j.1600-0404.2004.00351.x  10. Lublin FD, Cofield SS, Cutter GR, Conwit R, Narayana PA, Nelson F, et al. Randomized study combining interferon and glatiramer acetate in multiple sclerosis. Ann Neurol. 2013 Mar;73(3):327–40.  11. Lublin FD, Cofield SS, Cutter GR, Gustafson T, Krieger S, Narayana PA, et al. Long-term follow-up of a randomized study of combination interferon and glatiramer acetate in multiple sclerosis: Efficacy and safety results up to 7 years. Mult Scler Relat Disord [Internet]. 2017;18:95–102. Available from: https://www.scopus.com/inward/record.uri?eid=2-s2.0-85030242971&doi=10.1016%2Fj.msard.2017.09.012&partnerID=40&md5=3ed0175f33e0eb2dd53e9c2302f95218  12. Gold R, Arnold DL, Bar-Or A, Hutchinson M, Kappos L, Havrdova E, et al. Long-term effects of delayed-release dimethyl fumarate in multiple sclerosis: Interim analysis of ENDORSE, a randomized extension study. Mult Scler [Internet]. 2017 Feb;23(2):253–65. Available from: http://www.ncbi.nlm.nih.gov/pubmed/27207449  13. Kappos L, O’Connor P, Radue E-W, Polman C, Hohlfeld R, Selmaj K, et al. Long-term effects of fingolimod in multiple sclerosis: the randomized FREEDOMS extension trial. Neurology. 2015 Apr;84(15):1582–91.  14. Khan O, Rieckmann P, Boyko A, Selmaj K, Ashtamker N, Davis MD, et al. Efficacy and safety of a three-times-weekly dosing regimen of glatiramer acetate in relapsing-remitting multiple sclerosis patients: 3-year results of the Glatiramer Acetate Low-Frequency Administration open-label extension study. Mult Scler. 2017 May;23(6):818–29.  15. Moccia M, Palladino R, Carotenuto A, Saccà F, Russo CV, Lanzillo R, et al. A 8-year retrospective cohort study comparing Interferon-β formulations for relapsing‐remitting multiple sclerosis. Mult Scler Relat Disord [Internet]. 2018 Jan;19:50–4. Available from: https://www.scopus.com/inward/record.uri?eid=2-s2.0-85033384902&doi=10.1016%2Fj.msard.2017.11.006&partnerID=40&md5=b2d4e974b28c0eee8e9abe677a54ee9e  16. Onesti E, Bagnato F, Tomassini V, Volante G, Denaro F, Frontoni M, et al. Interferon beta treatment of MS in the daily clinical setting: a 3-year post-marketing study. Neurol Sci Off J Ital Neurol Soc Ital Soc Clin Neurophysiol. 2003 Dec;24(5):340–5.  17. Freedman MS, Francis GS, Sanders EACM, Rice GPA, O’Connor P, Comi G, et al. Randomized study of once-weekly interferon beta-1la therapy in relapsing multiple sclerosis: three-year data from the OWIMS study. Mult Scler. 2005 Feb;11(1):41–5.  18. Patti F, Pappalardo A, Florio C, Politi G, Fiorilla T, Reggio E, et al. Effects of interferon beta-1a and -1b over time: 6-year results of an observational head-to-head study. Acta Neurol Scand. 2006 Apr;113(4):241–7.  19. PRISMS-4: Long-term efficacy of interferon- -1a in relapsing MS. Neurology [Internet]. 2001 Jun 26;56(12):1628–36. Available from: http://www.neurology.org/cgi/doi/10.1212/WNL.56.12.1628  20. Gold R, Rieckmann P, Chang P, Abdalla J. The long-term safety and tolerability of high-dose interferon beta-1a in relapsing-remitting multiple sclerosis: 4-year data from the PRISMS study. Eur J Neurol [Internet]. 2005/08/02. 2005 Aug;12(8):649–56. Available from: http://doi.wiley.com/10.1111/j.1468-1331.2005.01083.x  21. Oger J, Francis G, Chang P. Prospective assessment of changing from placebo to IFN beta-1a in relapsing MS: the PRISMS study. J Neurol Sci. 2005 Oct;237(1–2):45–52.  22. Rio J, Tintore M, Nos C, Tellez N, Galan I, Montalban X. Interferon beta in relapsing-remitting multiple sclerosis. An eight years experience in a specialist multiple sclerosis centre. J Neurol. 2005 Jul;252(7):795–800.  23. Ruggieri RM, Settipani N, Viviano L, Attanasio M, Giglia L, Almasio P, et al. Long-term interferon-beta treatment for multiple sclerosis. Neurol Sci Off J Ital Neurol Soc Ital Soc Clin Neurophysiol. 2003 Dec;24(5):361–4.  24. Saida T, Itoyama Y, Kikuchi S, Hao Q, Kurosawa T, Ueda K, et al. Long-term efficacy and safety of fingolimod in Japanese patients with relapsing multiple sclerosis: 3-year results of the phase 2 extension study. BMC Neurol. 2017 Jan;17(1):17.  25. Cohen JA, Khatri B, Barkhof F, Comi G, Hartung H-P, Montalban X, et al. Long-term (up to 4.5 years) treatment with fingolimod in multiple sclerosis: results from the extension of the randomised TRANSFORMS study. J Neurol Neurosurg Psychiatry. 2016 May;87(5):468–75. |
| --- |
