# Supplementary material for: Reliability in long-term clinical studies of disease-modifying therapies for relapsing-remitting multiple sclerosis: A systematic review
Source: PLoS One. 2020 Jun 16;15(6):e0231722. doi: 10.1371/journal.pone.0231722 (PMC7297314; doi:10.1371/journal.pone.0231722)
Supplement: Attachment — Submitted filename: Response to Reviewers.docx [file pone.0231722.s002.docx]

Dear Aristeidis H. Katsanos,

We would like to thank you and the reviewers for the constructive review and for the suggestions for improving our manuscript for publication in Plos One.

We send below the responses and comments to the editor and reviewers:

**Editor**: Thank you for submitting your manuscript to PLOS ONE. After careful consideration, we feel that it has merit but does not fully meet PLOS ONE’s publication criteria as it currently stands. Therefore, we invite you to submit a revised version of the manuscript that addresses the points raised during the review process.

Both Reviewer's suggested that the manuscript should be more clinically oriented, appealing to the general neurology audience with no expertise in trial design or statistics.

**Authors:** We appreciate the consideration and analyze the discussion to add more clinical implications and, in addition, we add a clear recommendation to the clinician. In addition, we address other concerns highlighted by the reviewers (all changes are highlighted in blue in the text). Finally, both in the text and in the system, we have corrected institutional information about one of the authors and information about competing interests, as follows: "RL reports personal fees from Biogen and Roche; JB reports grants and personal fees from Biogen, Novartis, Roche and Teva and personal fees from Bayer, Ipsen, Merck Serono, Sanofi, outside of the submitted work. LL, MG, RP, FFL and AW declare that they have no conflict of interest. This does not alter our adherence to PLOS ONE policies on sharing data and materials. This funder had no role in any of the study phases (ie study design, data collection, data analysis, interpretation, report writing and submission responsibility) ".

**Comments to the Author**

5. Review Comments to the Author

**Reviewer #1:** This a well written metanalysis of long term open label DMT studies in MS.The statistical analysis is more than adequate. The authors conclude that these studies have low quality and clinicians should not rely on them for clinical decisions. Some points to be addressed : In the abstract, the authors state that "peginterferon, alemtuzumab, and cladribine are the most effective DMT considering ARR.:" Since all these studies have numerous biases and since some DMTs (natalizumab, ocrelizumab and teriflounomide) were not studied in this metanalysis, this statement may be misleading to the readers and would better be avoided. In addition , it is not clearly supported in the main body of the manuscript. In fact , authors rely on tables and do not fully discuss their findings in the main text.

**Authors**: We fully agree with the reviewer and the abstract has been revised (excerpts from the abstract are highlighted).

**Reviewer #1:** Lines 172-175 : DMTs and doses studied are presented in abbreviations that are not described in text, One has to search to the tables in order to clarify which abbreviation is which DMT. The authors should rephrase these sentences in a way that readers understand the sabstances mentioned.
**Authors**: We fixed it (excerpts from the methods are highlighted).

**Reviewer #1:** In general, the authors empasize on findings of the programs used in this metanalysis. This makes the manuscript sound quite " technical" and not clinician friendly . Some recommendations for clinicians could be also added in the text.

**Authors**: We agree. The discussion has been revised and the changes are highlighted.

**Reviewer #2:** Congratulations for your systematic review and the meaningful approach to a hot issue, such as clinical management of MS patients. I think that your work confirmed the fact that mid-term as well as long-term studies should be evaluated with criticism from the clinician perspective, because of the risk of bias that is so high lighted in your paper, though all of those "limitations" in comparing studies are known barriers in clinical decision making. Thus, multiple sclerosis treatment guidelines, support their recommendations based on evidence from RCTs and long term studies, as you mentioned in your paper, but they are just recommendations providing some guidance to non experts neurologists in MS, they do not substitute clinical judgement nor provide personalised medicine practice.

**Authors:** We fully agree with the reviewer and add that consideration to the personalized medicine in the discussion and conclusion.

**Reviewer #2:** My overall impression of the paper was that it was more focused to the difficulties of achieving the aims of the study than the report of efficacy and safety outcomes. It was clear enough to me that the published longterm data are not reliable enough to inform clinical decision making, but my estimation is, that is something that is already known in the clinical setting. I would prefer the paper to be more clinical friendly-oriented and provide some light in the chaotic MS landscape.

**Authors**: In fact, concluding about the efficacy and safety of therapies considering the high methodological limitation of the studies is neither possible nor recommended, with the risk of suggesting that clinicians make their decisions based on the clinical findings identified here. On the other hand, we agree that some light should be given to clarify to clinicians the practical implications of these limitations. Therefore, the discussion was revised to incorporate these aspects and practical recommendations (highlighted excerpts).
