# Supplementary material for: Reliability in long-term clinical studies of disease-modifying therapies for relapsing-remitting multiple sclerosis: A systematic review
Source: PLoS One. 2020 Jun 16;15(6):e0231722. doi: 10.1371/journal.pone.0231722 (PMC7297314; doi:10.1371/journal.pone.0231722)
Supplement: Attachment — Submitted filename: Response to Reviewers2.docx [file pone.0231722.s003.docx]

Dear Aristeidis H. Katsanos,

We would like to thank you and the reviewers for the constructive review and for the suggestions for improving our manuscript for publication in Plos One.

We send below the responses and comments to the editor and reviewers:

**Comments to the Author**

6. Review Comments to the Author

Reviewer #1: (No Response)

Reviewer #2: Thank you for your revision. There are some extra minor revisions that you should make. lines 52-54 needs rephrasing, lines 229-232 needs rephrasing, lines 323-325 needs rephrasing. Personalized medicine is not patient's experience in terms of effectiveness and safety. Please see personalized medicine in multiple sclerosis, and the definition of personalized medicine and then rephrase the lines mentioned above. All other corrections were ok

Authors: Thanks for the comment. We reviewed the three excerpts (highlighted), considering publications by Giovannoni 2017 and Pellegrini et al. 2019.
